# Glutamyl Endopeptidases: The Puzzle of Substrate Specificity

**Published:** 2017

**Authors:** I.V. Demidyuk, K.N. Chukhontseva, S.V. Kostrov

**Affiliations:** Institute of Molecular Genetics, Russian Academy of Sciences, Kurchatov Sq., 2, Moscow, 123182, Russia

**Keywords:** 3C-like serine protease, chymotrypsin-like protease, epidermolytic toxin, glutamyl endopeptidase, substrate specificity, V8 protease

## Abstract

Glutamyl endopeptidases (GEPases) are chymotrypsin-like enzymes that preferentially cleave the peptide bonds of the α-carboxyl groups of glutamic acid. Despite the many years of research, the structural determinants underlying the strong substrate specificity of GEPases still remain unclear. In this review, data concerning the molecular mechanisms that determine the substrate preference of GEPases is generalized. In addition, the biological functions of and modern trends in the research into these enzymes are outlined.

## GLUTAMYL ENDOPEPTIDASES AS MEMBERS OF THE STRUCTURAL CHYMOTRYPSIN FAMILY

**Fig. 1:**
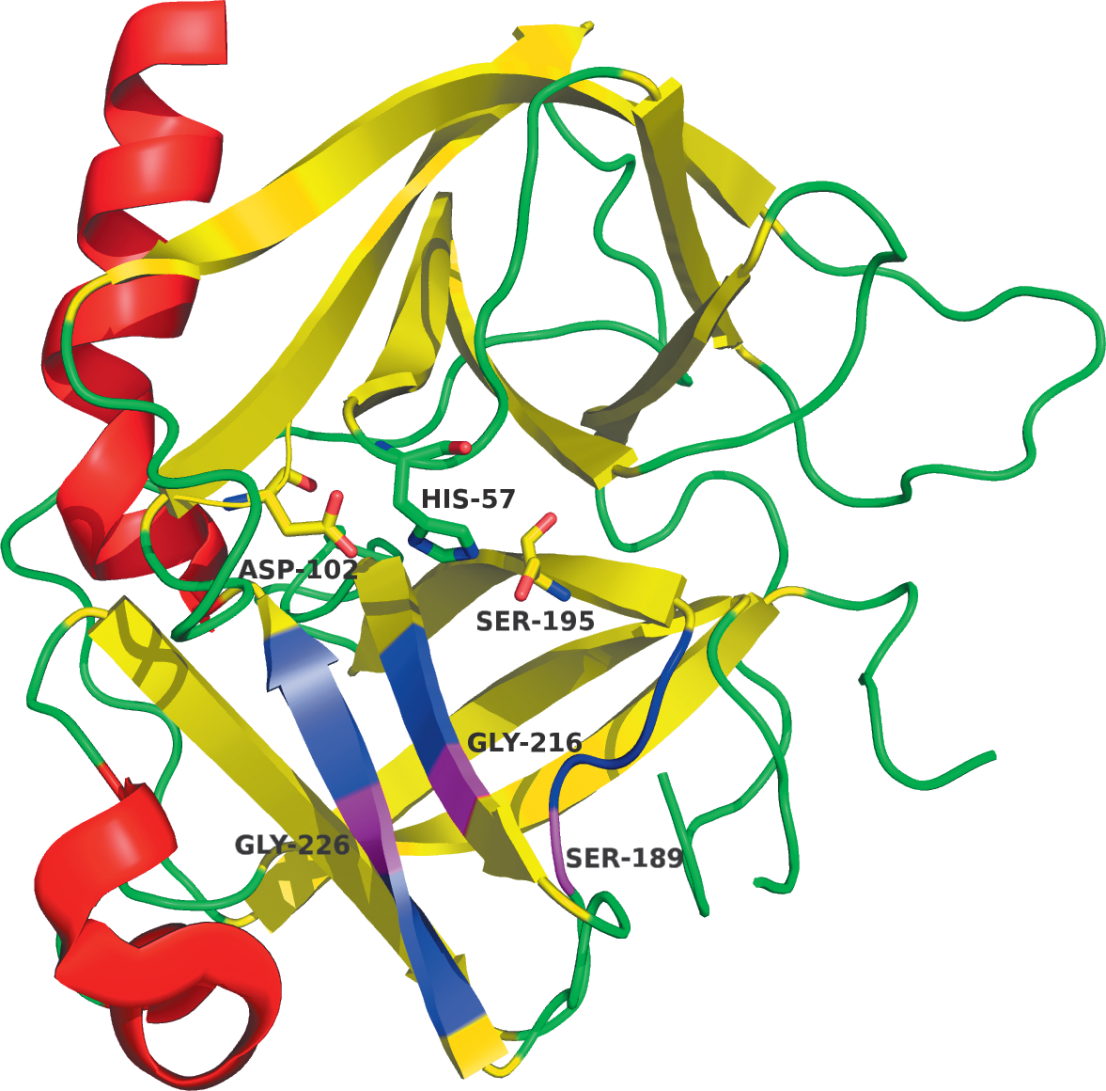
Three-dimensional structure of chymotrypsin (PDB ID – 5cha). The catalytic triad residues are shown as sticks. The regions forming the S1 pocket are shown in blue; the positions of the key residues of the S1 pocket are shown in magenta. All 3D structure pictures were generated using the PyMOL Molecular Graphics System (www.pymol.org).

Glutamyl endopeptidases (GEPases) are enzymes that preferentially cleave the bonds of the α-carboxyl groups of glutamic acid [1, 2]. GEPases from a number of gram-positive bacteria [23-25] and (+)RNA viruses have been characterized to date. All GEPases belong to the structural chymotrypsin family, which is one of the most extensive and well-studied families. Chymotrypsin-like protease (CLP) molecules share their spatial organization principle; the so-called chymotrypsin (or trypsin) fold (*Fig. 1*). The residue at the P1 position is a key determinant of the hydrolysis sites of CLPs (according to the Schechter and Berger nomenclature, the cleaved bond of the substrate is located downstream of the P1 residue, which corresponds to the S1-binding site of an enzyme [26]). Similar to pancreatic serine proteases, CLPs are conventionally classified into three main groups: 1) hydrolyzing bonds formed by the α-carboxyl groups of large hydrophobic amino acid residues (chymotrypsin-like specificity), 2) cleaving bonds downstream of positively charged residues (trypsin-like specificity), and 3) preferring small hydrophobic residues at the P1 position (elastase-like specificity) [27]. Furthermore, CLPs with mixed specificity have been discovered. For example, collagenolytic enzymes isolated from crabs exhibit the combined specificity of trypsin, chymotrypsin, and elastase [28], while bovine duodenase [29] and cathepsin G [30] can efficiently hydrolyze the substrates of both trypsin and chymotrypsin. In addition, CLPs cleaving bonds preferentially downstream of the Gln residue (e.g., many 3C-like viral proteases [23]) and being specific to negatively charged amino acid residues (e.g., granzyme B that preferentially hydrolyzes bonds downstream of Asp residues [31] and the GEPases that this review focuses on) are known.

CLP molecules consist of two perpendicular β-cylindrical domains and a C-terminal α-helix (*Fig. 1*). The catalytic and substrate-binding sites reside in the cleft between the two β-cylinders. The functionally important residues are predominantly localized in the loops connecting the β-strands. The S1 pocket lying next to the catalytic residue Ser(Cys)195 (hereinafter, chymotrypsin numbering is used) is formed by the regions 189–192, 214–216, and 224–228. In most cases, the residues at positions 189, 216, and 226 are the key determinants of substrate specificity [32, 33]. The enzymes capable of recognizing charged residues at position P1 carry residues compensating for the substrate charge at position 189 (Asp in trypsin [34]) or 226 (Arg in granzyme B [35], Glu in cathepsin G [36], and Asp in crab collagenase [37] and duodenase [38]). This gives grounds for believing that the primary substrate specificity of CLPs is controlled by a relatively small number of structural elements of the S1 site. However, the substrate specificity cannot be “switched” by just transferring these structural elements from one molecule into another.

As it has been demonstrated for the conversion of trypsin to chymotrypsin, specificity is also affected by a combination of remote structural elements that do not directly interact with the substrate. The S1 sites are similar in both enzymes. However, substitution of the main determinant of the binding of the charged substrates of trypsin Asp189 with Ser, which is typical of chymotrypsin, does not induce the corresponding specificity. Instead, a low-efficiency nonspecific protease is formed [39]. Ensuring chymotrypsin-like specificity requires substitution of four residues in the S1 pocket and modification of the regions remote from the S1 site: two surface loops that do not come into direct contact with the substrate [40] and Tyr172 residue [41]. Comparison of the crystalline structures and kinetic characteristics of the resulting variants to those of chymotrypsin and trypsin demonstrates that additional modifications are important for accurate positioning of the bond being cleaved with respect to the catalytic center of the protein (the Ser195–His57 pair and the oxyanion hole) rather than for binding the P1 residue [40-43].

Hence, according to the data on the structural determinants of the substrate specificity of CLPs, one can expect that the preference of negatively charged amino acid residues at the P1 position by GEPases is determined by the same regions of the polypeptide chain as in other enzymes belonging to this group. The substrate charge compensator is expected to be the key structural determinant of specificity, as well as in all the CLPs recognizing charged P1 residues. Arg or Lys at position 189 or 226 can be suggested as candidates for this. Meanwhile, one should bear in mind that the structure of the regions remote from S1 plays a significant role in high-efficiency interaction with the P1 residue.

## GLUTAMYL ENDOPEPTIDASE FROM STREPTOMYCES GRISEUS

**Fig. 2:**
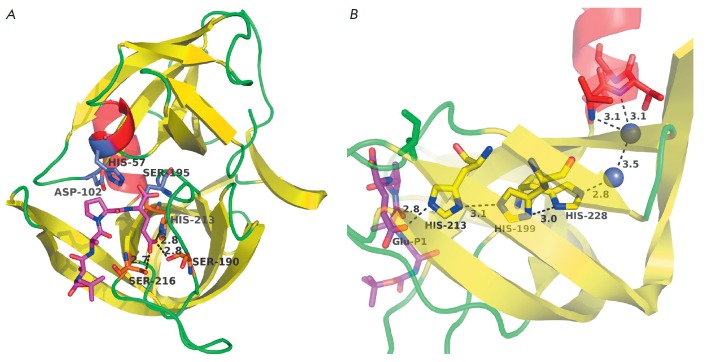
Three-dimensional structure of the glutamyl endopeptidase of *Streptomyces griseus *(1hpg). A – general view. B – the histidine triad. The Boc-AAPE ligand (the structure of the protecting group is not shown) is colored in magenta; the catalytic triad residues, in blue; the residues directly interacting with the carboxyl group of Glu-P1, in orange; and the histidine triad, in yellow. Water molecules are represented as blue spheres. The distances are given in angstroms.

Glu-specific protease from *S. griseus *(Glu-SGP) (PDB ID – 1hpg) was the first GEPase whose spatial structure was determined [44]. The structure of this enzyme is generally typical for CLPs (*Fig. 2A*) and is the most similar to that of bacterial CLPs (proteases A and B from *Str. griseus *and α-lytic protease). The overall geometry of the S1 site is also very close to the geometry of this region in the aforelisted bacterial enzymes. Contrary to expectations, no explicit compensator for the negative charge of the substrate, Lys or Arg residue, was detected in the S1 site. The carboxyl group of Glu at position P1 of the substrate forms hydrogen bonds with Ser190 (192 if numbering [44] is used), Ser126, and His213. Hence, these residues probably play the key role in substrate recognition. The side chain of histidine can be positively charged. However, if pKa of the side chain of His213 in the absence of the substrate is taken to be 6.4, the imidazole ring will be protonated by less than 1% at the pH 8.5 that is optimum for the functioning of Glu–SGP; therefore, histidine is expected to be neutrally charged [44]. Meanwhile, pKa of amino acid residues in the proteins can vary significantly depending on the environment [45]. An analysis of the Glu-SGP structure has revealed that it carries the so-called histidine triad containing His199 and His228, along with His213. The three His residues permeate the C-terminal β-cylindrical domain to form a chain of hydrogen bonds that links the carboxyl group of the substrate Glu–P1 and, via two water molecules bound to the enzyme, the N-terminal rim of the C-terminal α-helix of the molecule (*Fig. 2B*). It was postulated that this very structure ensures the transfer of the positive charge compensating for the substrate charge from the microdipole of the α-helix to His213 of the substrate-binding site [44]. Let us mention that the histidine triad residues in GEPases are not conserved [46] and, in addition to Glu–SGP, have been found only in the highly homologous enzyme from *Str. fradiae *[13].

The role of the residues forming the S1 pocket and the histidine triad Glu-SGP was investigated by site-directed mutagenesis. Any modifications to Ser190(192) (Ala/Gly/Asn/Thr/Val) and His213 (Ala/Gly/Lys/ Asn/Arg/Ser/Val) stop the autocatalytic processing (at Glu(-1)–Val1 bond) of the GEPase precursor, which proves that these residues play a fundamental role in the formation of the S1 site. Meanwhile, Ser216 seems to be less important, since its substitution for Ala or Gly does not result in a loss of activity by the enzyme. A similar result was observed for certain modifications of histidine triad residues: the mutations His199→Val and His228→Ala/Asp/Asn/Ser/Val do not impede enzyme processing. All the mutant proteins (His199→Val, Ser216→Ala, Ser216→Gly, and His228→Ala) whose specificities have been studied maintained their preference for the substrates carrying Glu-P1 [47]. Hence, the hypothesis of the significance of the histidine triad in charge compensation has not been confirmed experimentally and the Ser190(192) and His213 residues are now believed to play a key role in substrate recognition.

Thus, while the structure of the S1 site is already known, it remains unclear how the elements forming this site can ensure the observed substrate specificity. This controversy remains even more explicit once the data on the structure and specificity of viral 3C-like serine proteases are examined.

## VIRAL 3C-LIKE SERINE PROTEASES

Processing of polyprotein precursors is an integral part of the life cycle of most (+)RNA viruses [48-50] and typically involves viral papain-like or chymotrypsin-like proteases, components of the polyprotein [51]. Most CLPs from (+)RNA viruses are cysteine proteases, such as 3C proteases (3Cpro) of picornaviruses or 3C-like proteases (3CLpro) of corona-, poty-, or comoviruses [49]. Meanwhile, some enzymes whose active sites contain the serine catalytic residue have been identified. These proteins are denoted as 3C-like serine proteases (3CLSP) [23]. CLPs from (+)RNA viruses exhibit a narrow substrate specificity. The hydrolysis sites of the 3C and 3C-like proteases are generally similar and usually contain a Gln or Glu residue at the P1 position along with a small amino acid residue located downstream (Gly, Ala, or Ser) [23, 52]. Some proteases cleave the bonds formed by both Gln and Glu [53-57], while others prefer Gln-P1 (e.g., 3Cpro or 3CLpro of picornaviruses and coronaviruses [23, 48]) or are true GEPases cleaving the polypeptide chain right after Glu. Such specificity is exhibited by CLPs of arteri-[23], sobemo- [25], and astroviruses [24].

**Fig. 3:**
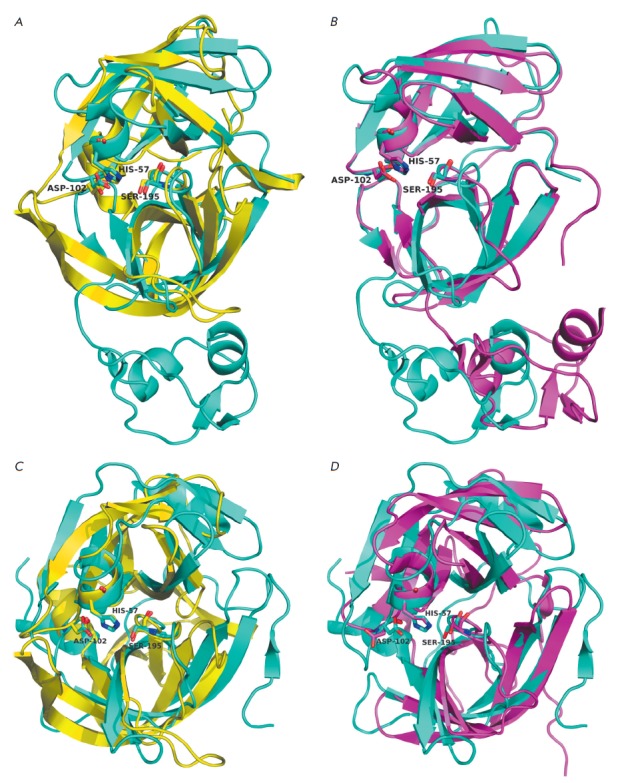
Three-dimensional structures of viral glutamyl endopeptidases. A - EAVnsp4 (PDB ID – 1mbm; blue) and GluSGP (1hpg; yellow). B – EAVnsp4 (cyan) and PRRSV-nsp4 (3fan; magenta). C – GluSGP (yellow) and SeMV-pro (1zyo; cyan). D – SeMVpro (cyan) and HAstV-pro (2w5e; magenta). The catalytic triad residues are designated.

Arteriviral GEPases denoted as Nsp4 (nonstructural protein 4) [23] are serine proteases [58, 59]. Their properties have been studied, and the spatial structures of Nsp4 of the equine arteritis virus (EAV) and porcine reproductive and respiratory syndrome virus (PRRSV) have been identified. The 3D structure of EAV-Nsp4 is generally typical of CLPs (PDB ID – 1mbm). Meanwhile, the catalytic domain of the enzyme formed by two perpendicular β-cylinders also has a C-terminal extension (*Fig. 3A*) [60]. The structure of PRRSV-Nsp4 (PDB ID – 3fan) is similar to that of EAV-Nsp4; however, it noticeably differs in the mutual arrangement of the catalytic and C-terminal domains (*Fig. 3B*) [59].

**Fig. 4:**
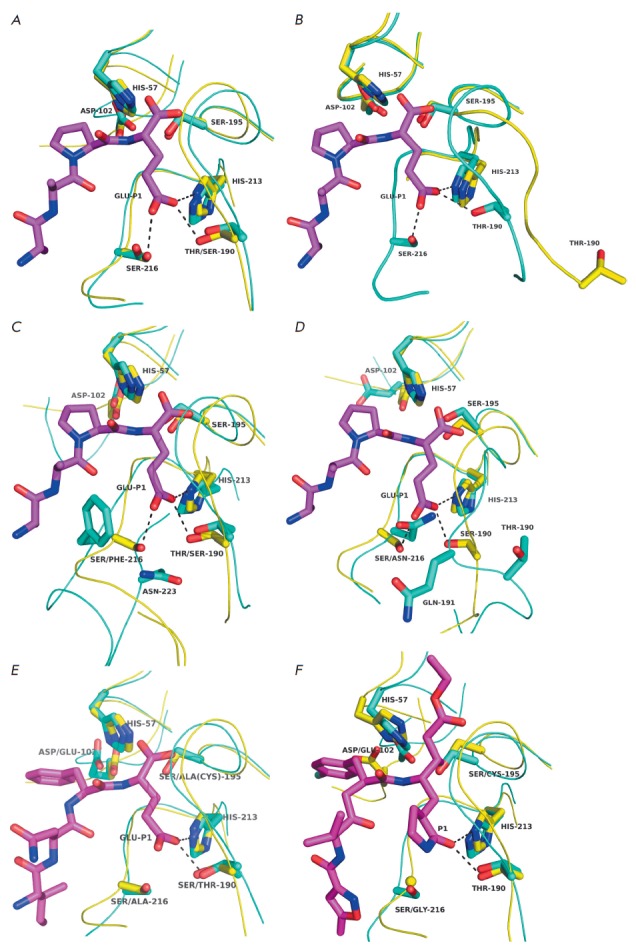
S1 sites of viral Glu– and Gln–specific proteases. A – EAV-nsp4 (1mbm; cyan) and Glu-SGP (1hpg; yellow) complexed with Boc-AAPE (magenta; the structure of the protecting group is not shown). B – EAV-nsp4 (cyan) and PRRSV-nsp4 (3fan; yellow); the Boc-AAPE from the Glu-SGP structure (magenta) is inserted into the S1 site. C – Glu-SGP (yellow) complexed with Boc-AAPE (magenta) and SeMV-pro (1zyo; cyan). D-Glu-SGP (yellow) complexed with Boc-AAPE (magenta) and HAstV-pro (2w5e; cyan). E – Glu-SGP (yellow) and Gln/Glu-specific Norwalk virus protease (4in1; cyan) with Cys195→Ala substitution complexed with tetrapeptide Ile-Asn-Phe-Glu (magenta). F – EAV-nsp4 (cyan) and Gln-specific human rhinovirus 3C protease (1cqq, yellow) complexed with inhibitor AG7088 (magenta). Dashed lines represent hydrogen bonds.

The architectures of the S1 sites of EAV-Nsp4 and Glu-SGP are very similar (*Fig. 4A*). The S1 pocket contains the same three main structural elements: His213 (134 in EAV-Nsp4, 1198 in polyprotein), Thr190 (115, 1179) corresponding to Ser190 in Glu-SGP, and Ser216 (137, 1201) [60]. All three residues are also found in the primary structure of PRRSV-Nsp4 [58, 59]. However, the crystal structure analysis data show that the S1 site of the latter enzyme has a structure different from those of EAV-Nsp4 and Glu-SGP (*Fig. 4B*). The position of the polypeptide chain region 190–194 (113–117 in PRRSV-Nsp4) is altered compared to that in most CLPs, resulting in a nontypical configuration of the oxyanion hole and a significant distance between Thr190(113) and the carboxyl group of Glu-P1. Furthermore, the position of the Ser216-containing loop 216–220 (136–140) could not be detected by a crystal structure analysis, thus demonstrating that this region is highly flexible. The arrangement of the most conserved residue in the S1 site, His213(133), in the aforementioned three proteins is identical [59]. This situation probably does not describe the state of PRRSV-Nsp4 in the solution but is an artifact of free-enzyme crystallization.

The importance of the His213 and Thr190 residues for the functioning of EAV-Nsp4 was confirmed using site-directed mutagenesis experiments. It was demonstrated by modifying the catalytic triad residues that processing of the polyprotein involving cleavage of the bonds after Glu residues depends on the activity of EAV-Nsp4. The modifications His213(1198)→Lys/Arg/ Tyr also terminated the processing. The same effect was observed with the Thr190(1179)→Asp substitution; however, the mutations Thr190(1179)→Ser/Gly only slightly reduced the processing efficiency [58]. In combination with the data obtained using the Glu–SGP model, these results demonstrate the fundamental significance of His213 and the considerably smaller role of the residues 190 and 216 for the hydrolysis of specific substrates by GEPases. Meanwhile, it still remains unclear whether His213 is a key element in the recognition of the charged substrate and what contribution to the formation of substrate specificity is made by Thr/ Ser190 and Ser216. An analysis of the structures of other viral GEPases will shed more light on some of these questions.

Sesbania mosaic virus protease (SeMV-pro) has a 3D structure typical of CLPs (PDB ID – 1zyo) that is more similar to those of cellular (in particular, Glu– SGP) rather than viral representatives of this family (*Fig. 3C*) [61]. The protease carries the conventional catalytic triad; modification of its residues terminates polyprotein processing [62]. Similar to all GEPases, the conserved residues His213(298) and Thr190(279) are maintained, located within the S1 site of the enzyme. However, position 216(301) is occupied by a large hydrophobic residue, Phe (*Fig. 4C*). Superimposition of the 3D structures of SeMV-pro and Glu-SGP complexed with the tetrapeptide product of proteolysis of *tert*-butyloxycarbonyl-Ala-Ala-Pro-Glu (Boc-AAPE) demonstrates that the side chain of the Glu–P1 residue fits well the S1 pocket of viral protease. In order for the volume of the S1 pocket to be retained if there is a residual with a bulky side chain, the main protein chain needs to be significantly shifted in the 214(299)– 223(308) region and the resulting space needs to be filled with the side chain of the Asp223(308) residue that is involved in the formation of the bottom of the S1 pocket, but apparently does not directly interact with Glu-P1 (*Fig. 4C*). This situation demonstrates that Ser 216 and the hydrogen bond between residue 216 and the γ-carboxyl group of Glu–P1 play no role in ensuring glutamate specificity. Unfortunately, no experiments involving the modification of Phe216(301) within SeMV–pro have been carried out. Meanwhile, the substitutions of His213(298) and Thr190(279) for Ala, but not the Asp223(308)→Ala mutation, completely inhibit the processing *in cis *of the SeMV-pro/VPg fusion protein (VPg being the viral protein following SeMV-pro in the polyprotein) in the model system [61].

The substrate specificity of human astrovirus protease (HAstV-pro) has been poorly studied. There is a lack of consistency in the data on the processing sites of viral polyprotein performed by this enzyme [63]. Meanwhile, it was demonstrated by using a recombinant enzyme and a series of synthetic substrate *in vitro *that HAstV-pro cleaves only the bonds formed by the α-carboxyl groups of Glu and Asp [24]. The spatial structure of HAstV-pro (PDB ID – 2w5e) is generally similar to that of SeMV-pro (*Fig. 3D*) but has a number of specific features. Hence, the Asp102 residue (489 in polyprotein) of the catalytic triad that also contains Ser195(551) and His57(461) possesses a noncanonical conformation [24].

The structure of the S1 site also noticeably differs from the ones discussed above. Despite the fact that the His213 residue and its position are invariant, Ser at position 216 is substituted by Asn216(569), whose amide group actually occupies the place of the γ-carboxyl group of the substrate Glu-P1 as demonstrated by the superposition of the HAstV-pro structure and Glu-SGP complexed with the ligand (*Fig. 4D*). This significantly reduces the S1 pocket [24], whose volume does not match the Glu side radical. Furthermore, the conformation of the main-chain region 189–193 (545–549) differs from that in most CLP; thereafter, the conserved Thr190 residue lies far from the S1 site and is turned sideways. The position of the region 189–193 resembles the configuration of this region in PRRSV-Nsp4. Taking into account these differences from the structures of other GEPases and CLPs, it is rather arduous to draw any specific conclusions regarding the interactions between HAstV-pro and the P1 residue of the substrate.

Having summarized the data on viral GEPases and Glu–SGP, one can draw a conclusion that His213 is the shared element of the S1 pocket, while its modification causes enzyme inactivation in most cases. This residue can be positively charged; therefore, it is regarded as a candidate for being the key structural element that determines the substrate preferences of Glu-specific proteases. The Thr/Ser190 residue is also conserved in all GEPases, but its modification does not result in a loss of specific activity by the enzymes and possibly does not play any crucial role in the recognition of the Glu- P1 residue of the substrate. Finally, the nature of residue 216 is unessential in ensuring substrate specificity. As a result, GEPases carry residues with strongly different properties, Ser, Asn and Phe, at these positions. Additional information on the structural determinants of the substrate specificity of GEPases can be obtained by analyzing the viral 3C and 3C-like proteases that exhibit specificity to Gln at position P1.

Comparison of the primary and spatial structures of GEPases and 3C/3CLpro shows the similarity between their S1 sites (*Fig. 3 E,F*). First, all 3C/3CLpro, identically to GEPases, contain the conserved His213 residue [64-76], whose modification results in enzyme inactivation [77-79]. This fact allows one to infer that this residue is not the key determinant of recognition of the substrate charge but is fundamental in ensuring a correct geometry of the S1 site. Second, most 3C/3CLpros retain the Thr/Ser190 residue that is typical of GEPases [58], thus confirming the conclusion that it is crucial for the formation of an adequate geometry of the S1 pocket rather than for charge recognition. The third element of the S1 site of GEPases at position 216, in 3C/3CLpro, is typically replaced with Gly (*Fig. 4F*) and sometimes Ala (*Fig. 4E*) residues, which have not been found in the known GEPases. The latter fact provides grounds for speculation about the involvement of Ser216 in the compensation for the substrate charge in GEPases [60]. However, mutagenesis in the Glu–SGP model shows that the Ser216→Ala/Gly substitution does not make the substrates with Gln–P1 the preferred ones, although it increases efficiency in their hydrolysis [47]. Furthermore, the data on GEPases with Phe/Asn216 residues that have been discussed do not support these assumptions. It is worth mentioning another hypothesis that still remains unverified. Since all GEPases are serine proteases, while Gln-specific enzymes are cysteine proteases, it is fair to assume that the difference in their substrate specificity depends on catalytic residues.

Hence, none of the detected conserved structural elements of the S1 site of Glu-SGP and viral 3CLSP seems to determine the preference of these enzymes for the Glu residue at the P1 position of the substrate. Therefore, this specificity of the GEPases of viruses and *Streptomyces *is ensured by structural determinants that do not directly reside in the substrate-binding site. However, the conventional research method combining the 3D structure analysis, site-directed mutagenesis, and studying the catalytic properties of enzymes has not identified these determinants yet. Studies focused on bacterial GEPases seem more successful.

## STAPHYLOCOCCAL EPIDERMOLYTIC TOXINS

Staphylococci produce two types of GEPases: enzymes similar to V8 protease from *Staphylococcus aureus *(Glu-V8), which will be discussed below, and epidermolytic toxins (ETs). ETs are the key virulence factors responsible for the development of bullous impetigo and its generalized form, staphylococcal scalded skin syndrome, as well as similar animal diseases [80, 81]. The biological activity of ETs is associated with their ability to cleave with high specificity the Glu381-Gly bond in desmoglein 1, the desmosomal protein of cadherin type that mediates intercellular contacts (see more details in review [80]). In addition, ETs cleave the ester bonds formed by the carboxyl groups of Glu residues *in vitro *[82].

**Fig. 5:**
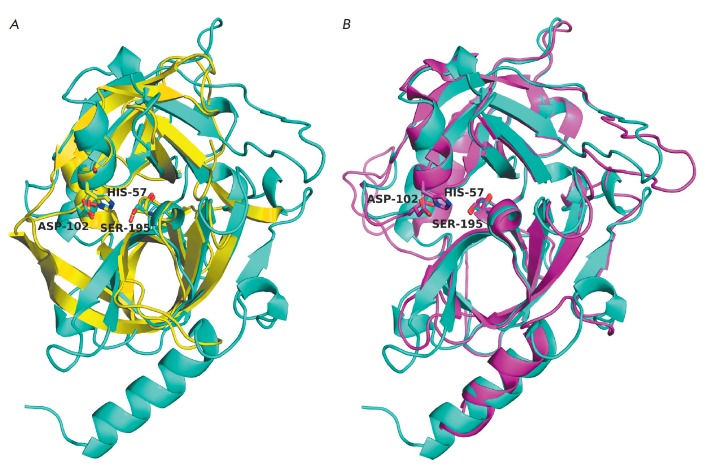
Three-dimensional structures of the epidermolytic toxins of *S. aureus*. A – ETA (1agj; cyan) and Glu-SGP (1hpg; yellow). B – ETA (cyan) and ETB (1qtf; magenta). The catalytic triad residues are designated.

The spatial structures of epidermolytic toxins A [83, 84] and B [85] from *S. aureus* demonstrate that ETs belong to the CLPs family (*Fig. 5*). Meanwhile, these proteins exhibit unique features, the N-terminal α-helix being one of them. The second feature consists in the unusual position of the residues forming the oxyanion hole: the Pro/Val192–Gly193 peptide bond (chymotrypsin numbering being used) is rotated 180° compared to other CLPs. As a result, a hydrogen bond is formed between the carbonyl oxygen of residue 192 and the hydroxyl group of catalytic Ser195 that seems to impede the manifestation of activity. After a structural analysis, a hypothesis has been put forward that binding between ET and the substrate (or a receptor) that the N-terminal α-helix is involved in results in a rearrangement of the active site and enzyme activation [83].

**Fig. 6:**
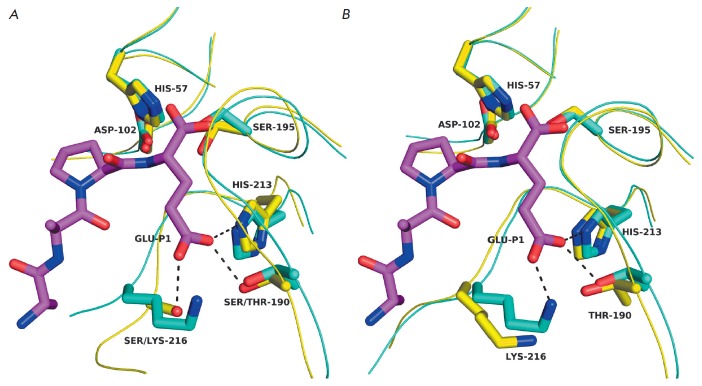
S1 sites of the epidermolytic toxins of *S. aureus*. A – ETA (1agj; cyan) and Glu-SGP (1hpg; yellow) complexed with Boc-AAPE (magenta; the structure of the protecting group is not shown). B – ETA (cyan) and ETB (1qtf, yellow); the Boc-AAPE from the Glu-SGP structure (magenta) is inserted into the S1 site. Dashed lines represent hydrogen bonds.

Identically to all the GEPases discussed above, the S1 pockets of ETs contain three key elements, two of which are the conserved His213 and Thr190 residues (*Fig. 6*). The third key element, as it has been predicted by simulation of the 3D structures [46], is Lys at position 216, which is an ideal candidate for compensating for the negative charge of Glu-P1 (Ser being typically found at this position in other GEPases). The Lys residue is conserved in most ETs from *S. aureus *and *S. hyicus*, while ExhA (an ET isolated from *S. hyicus*) contains Arg at position 216 [86, 87]. The significance of Lys216 for the hydrolysis of substrates containing the Glu residue has been confirmed by site-directed mutagenesis experiments performed for the ETA model [88]. Any of the Lys216→Ala/Glu/Thr substitutions, identically to mutations in residues of the catalytic triad, resulted in a loss of the protein’s ability to cleave N-Boc-*L*-glutamic acid α-phenyl ester and loss of epidermolytic activity.

Hence, in the case of ET, the positively charged residue that probably compensates for the substrate charge was detected directly in the S1 site, at position 216, which is important for substrate recognition by all CLPs. This compensator is critical for exhibiting enzymatic activity by ET. Meanwhile, there is no direct evidence yet that Lys/Arg216 in ET is responsible for glutamate specificity. The S1 sites of ET, except for Lys216, are very similar to the corresponding regions of GEPases from viruses and *Streptomyces *(*Fig. 6*). However, the findings presented above demonstrate that residue 216 is not significant in ensuring the substrate specificity of these enzymes. It should be inferred that different GEPase groups have different substrate recognition mechanisms. The standard charge compensator in the S1 pocket is the key structural element in ETs; in enzymes from viruses and *Streptomyces*, it is some other remote structural element. This conclusion has been supported by the data obtained for other bacterial GEPases.

## OTHER BACTERIAL GLUTAMYL ENDOPEPTIDASES

In addition to GEPases from *Streptomyces *and ETs, a number of proteases secreted by gram-positive bacteria and possessing common structural features have been characterized. The simulation of the 3D structures of enzymes belonging to this group (Glu-V8, GEPases from *Bacillus licheniformis *and *B. subtilis*) conducted at early stages of the study of GEPases produced the assumption that compensation of the substrate charge in all three proteins is ensured by the α-amino group of residue 1 in the mature enzyme [46]. Localization of the N-terminal residue in the S1 site of GEPases of this group was verified later by experimental data on the tertiary structures of GEPase from *B. intermedius *(BIGEP) [89], Glu-V8 [90], and extracellular serine protease from *S. epidermidis *(Esp) [91].

**Fig. 7:**
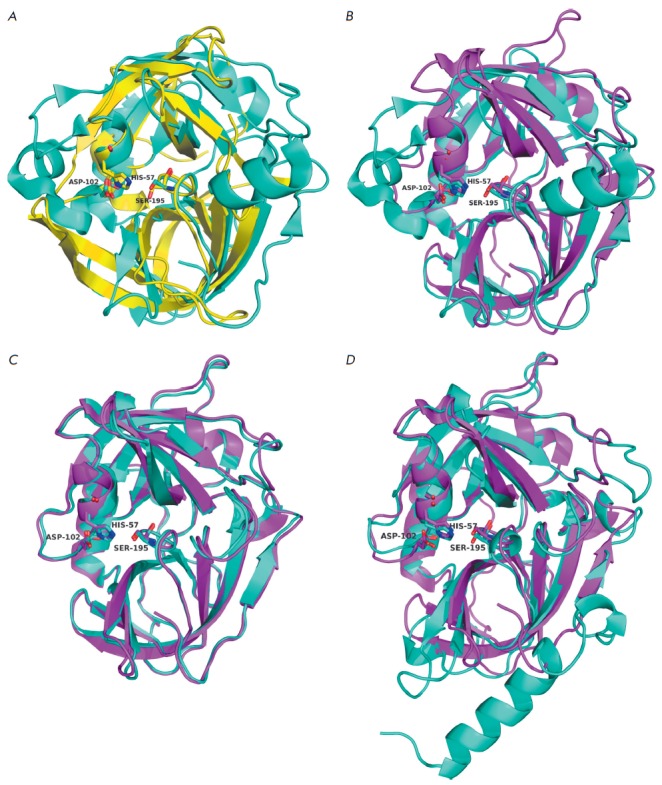
Three-dimensional structures of bacterial glutamyl endopeptidases. A – BIGEP (1p3c, cyan) and Glu-SGP (1hpg; yellow). B – BIGEP (cyan) and Glu-V8 (1qy6; magenta). C – Glu-V8 (magenta) and Esp (4jcn; cyan). D – Glu-V8 (magenta) and ETA *S. aureus *(1agj; cyan). The catalytic triad residues are designated.

**Fig. 8:**
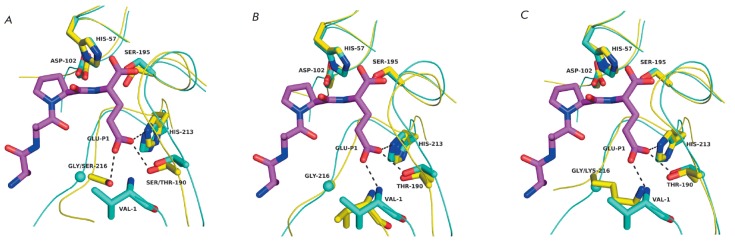
S1 sites of bacterial glutamyl endopeptidases. A – BIGEP (1p3c, cyan) and Glu-SGP (1hpg; yellow) complexed with Boc-AAPE (magenta; the structure of the protecting group is not shown). B – BIGEP (cyan) and Glu-V8 (1qy6, yellow). C – BIGEP (cyan) and ETA (1agj, yellow). In B and C, the Boc-AAPE from the Glu-SGP structure (magenta) is inserted into the S1 site. Dashed lines represent hydrogen bonds.

The proteins under discussion possess high structural similarity with each other and with staphylococcal ETs; their structure is typical of CLPs. Their molecules consist of two β-domains separated by a deep cleft containing the active site (*Fig. 7*). The general architecture of the S1 sites in Glu-V8, BIGEP, and Esp is similar to that of analogous regions in other GEPases and contain the mandatory elements: His213 and Ser/Thr190 (*Fig. 8 A,B*). Meanwhile, the Gly residue is located in the third key position of the S1 pocket, which is a feature of viral Gln-specific 3C– and 3CLpro as discussed above. However, the absence of residue 216 side radical that can form a hydrogen bond with the carboxyl group oxygen of the substrate is compensated for, as predicted earlier, by the α-amino group of Val1, which occupies a position corresponding to that of the ε-amino group of the Lys216 residue in ET (*Fig. 8C*). Hence, a unique situation seems to take place for Glu-V8, BIGEP, and Esp, when protease specificity is determined by the N-terminus of the polypeptide chain. The originality of this “design concept” consists in the fact that Glu-V8, BIGEP, and Esp are synthesized by the cell as precursors that involve the signal peptide and propeptide, in addition to the catalytic domain. Hence, the N-terminus of a mature protein and, therefore, the S1 pocket are formed only after processing. This situation resembles the mechanism of activation of mammalian CLPs: after the propeptide was removed, the N-terminal NH2-group of the mature protein formed a salt bridge with the Asp194 residue, thus triggering structural rearrangements in the enzyme molecule that result in its activation due to the formation of a proper structure of the S1 site and an oxyanion hole [92-96].

Site-directed modification of the residues in the S1 sites of BIGEP and Glu-V8 provided interesting results. First of all, the GEPase variant with a modification of the His213 residue was studied for the first time. Mutations of this type had been inserted earlier [47, 58], but no proteins were obtained. It was demonstrated that BIGEP with the His213(186 in BIGEP)→Thr substitution does not alter substrate preference and cleaves the protein substrate only after Glu residues. Meanwhile, modification significantly affects the catalysis effectiveness (the *k*_cat_ decreases more than 600-fold) but has a relatively low impact on substrate binding (the *K*_M_ increases approximately fivefold) [97]. Interestingly, a similar effect is also observed when the substrates containing the Asp residue at the P1 position are cleaved by native GEPases: the *K*_M_ increases approximately sixfold, while the *k*_cat_ declines by the same order of magnitude (~150-fold) [98]. These findings allow one to conclude that the conserved His213 residue is not the key element that determines the recognition of the negative charge of the substrate by GEPases but seems to be significant for accurate positioning of the cleaved bond with respect to the nucleophile (oxygen of the hydroxyl group of Ser195). This conclusion is consistent with the fact that His213 is the common structural element for Glu- and Gln-specific proteases.

The data on the role of the N-terminal residue in the functioning of GEPases were obtained for the Glu–V8 model. The substitution of the N-terminal Val for Leu/ Ala/Phe/Gly/Ser was shown to reduce the efficiency of hydrolysis of the substrates carrying the Glu residue approximately 3-, 20-, 50-, 100-, and 200-fold, respectively [9, 99]. The more properties of the residue are similar to those of Val, the smaller the decrease in activity is. This result indicates that residue 1 is important for enzyme function and can be explained by the fact that deviations of the position of the α-amino group of this residue from the optimal position are different in mutants. Furthermore, Glu-V8 variants with additional amino acid residues, propeptide fragments, at the N-terminus, have been successfully obtained. Insertion of additional residues (from 1 to 39) in all cases significantly reduced enzymatic activity in hydrolyzing the substrates containing Glu-P1 [9, 100] but had a smaller impact on the efficiency of hydrolysis of similar substrates carrying Gln-P1. The mutants maintained their preference for substrates containing Glu-P1; cleavage efficiency was 10–20 times higher [100]. Hence, the α-amino group of the N-terminal Val residue probably makes a very significant contribution to the recognition of the charged substrate by the bacterial GEPases under discussion but is not fully responsible for enzyme specificity.

Summarizing all the available data regarding GEPases, a conclusion can be drawn about the differences in the mechanisms of recognition of charged substrates by enzymes belonging to various groups. This indicates that GEPase branches have appeared several times in the evolutionary tree of CLPs, probably on the basis of the fundamental structure of the S1 pocket that is equally suitable for ensuring both glutamate and glutamine specificities and is most similar to the structure of the S1 regions of viral enzymes. The necessity for several structural variants of specificity optimization apparently is supposed to be caused by the differences in the functions of proteases belonging to different groups. An analysis of the published data on GEPases reveals that variations in the structure of the S1 sites in these enzymes correlate with the differences in the maturation mechanisms of their precursors. This observation allows one to put forward a hypothesis that the charge-compensation method depends on the maturation mechanism of the precursor protein.

## STRUCTURAL DETERMINANTS OF SUBSTRATE SPECIFICITY AND MATURATION OF GLUTAMYL ENDOPEPTIDASE PRECURSORS

All GEPases are synthesized as precursors. However, the enzyme processing mechanisms significantly differ and can be subdivided into three groups. GEPases from *Streptomyces *and viruses are processed autocatalytically [47, 51]. ET precursors contain only a secretory leader [3] and, therefore, are processed by signal peptidase. For bacterial GEPases similar to Glu-V8 and BIGEP, propeptide is removed heterocatalytically by different proteases [22, 100-104], with just one exception [21]. Comparison of the structures of the S1 sites of GEPases and the processing mechanisms shows that no explicit substrate charge compensator is revealed in the S1 pocket in autoactivated enzymes; the S1 site of ET is characterized by the presence of the Lys216 residue, while the GEPases similar to Glu–V8 and BIGEP processed heterocatalytically contain an α-amino group of the N-terminal residue. Let us discuss these matches in the context of the biological functions of proteases belonging to each group.

Viral GEPases are synthesized as part of the long polyprotein, its selective hydrolysis being the main function of these enzymes [25, 51, 105, 106]. Hence, viral GEPases function inside the cell and start acting immediately after the polyprotein is synthesized. Therefore, the active site of the enzyme, including the specificity-determining regions, needs to form and be able to perform high-specificity hydrolysis already as part of the precursor protein, maintaining its structure after processing. The function of GEPases from *Streptomyces *appears to be fundamentally different. These extracellular enzymes are synthesized as conventional protease precursors carrying prepropeptide. The functions of the prosequences of GEPases from *Streptomyces *are yet to be elucidated; however, one can assume that propeptides ensure the kinetic stability of mature molecules and partake in their secretion, by analogy with the closely related protease B from *Str. griseus *[107, 108]. Meanwhile, autocatalytic processing and the lack of a noticeable post-translational regulation of activity make this situation similar to that reported for viral enzymes: the active site needs to have completely formed within a precursor and maintained intact after a mature molecule has formed. In both cases, this problem seems to have one structural solution (*Fig. 4*). The S1 pocket does not have a direct charge compensator. The N-terminus is remote from the active site in the mature protein. Therefore, it does not partake in the formation of the S1 site as it is involved in processing. The structural elements responsible for glutamate specificity, which have not been identified yet, reside outside the S1 region and probably form before precursor processing. Hence, the structural elements that change during maturation are not involved in the formation of the molecule sites important for catalysis.

The opposite is observed for GEPases synthesized as preproprotein (e.g., Glu-V8). Not only are these proteases subjected to heteroactivation [101-103, 109], but they are also involved in regulatory activation cascades as it was demonstrated for Glu-V8 [110-112]. This implies that activity is strictly controlled via a rather complex and somewhat controversial mechanism. At first glance, the Glu–V8, BIGEP, and Esp precursors are supposed to be inactive, since the S1 site in these proteins is formed only in the mature molecule (*Fig. 7* and *Fig. 8*). Meanwhile, data have been published demonstrating that the precursors of Glu-V8 [113], BIGEP [109], Esp [7], as well as GEPases from *B. licheniformis *[114], *B. subtilis *[102], and *Thermoactinomyces *sp. [21] are capable of autoprocessing; in most cases, it is the bonds corresponding to the specificity of the mature enzymes that are cleaved [7, 21, 109, 114]. Furthermore, glutamate activity *in trans *of precursor analogues has been detected [100]. These facts cast doubt on the mere possibility of regulating the activity of the proteases under discussion, although a closer look at the precursor activation mechanism demonstrates that the situation is more complex.

Autoprocessing (maybe intramolecular) of native enzymes that spontaneously occurs both *in vitro *and *in vivo *results in the formation of protein species with propeptide fragments usually 3–15 a.a.r. long rather than in complete deletion of the prosequence [7, 100, 109, 113, 114] that corresponds to the size of propeptides in mammalian CLPs. These species exhibit no activity with respect to protein substrates and low activity with respect to peptides *in trans *and can be activated only heterocatalytically [7, 100, 109, 113, 114]. To make the picture complete, we would like to add that data on the enzyme from *Thermoactinomyces *sp. carrying Glu1, which can be autoactivated *in vitro *in a heterologous expression system [21], identically to the previously artificially obtained mutants of other GEPases [109, 114], have been published recently. Hence, maturation of enzymes similar to Glu–V8 is a stepwise process. These proteins seem to contain two propeptides. The first one is a long folding assistant [99, 109] that ensures the kinetic stability of a mature protein as often occurs in bacterial proteases [115]. The second propeptide, which is short and forms after the first processing step, is the activation unit [109, 113, 114] that maintains the inactive state of the enzyme. Furthermore, it cannot be ruled out that the structure of the active site of proteases changes after the first propeptide portion is removed. It is fair to say that propeptides of the discussed group of GEPases simultaneously combine properties typical of the propeptides of bacterial CLPs and mammalian enzymes. Hence, the need for strict regulation of the activity of Glu-V8-like enzymes is satisfied through the formation of the S1 pocket only after the propeptide has been deleted. A mechanism similar to the activation mechanism of mammalian CLPs is used: involvement of the N-terminal amino group in the structure of the molecule elements essential for catalysis. However, the folding assistant is deleted autocatalytically due to the basic specificity of the enzymes.

**Fig. 9:**
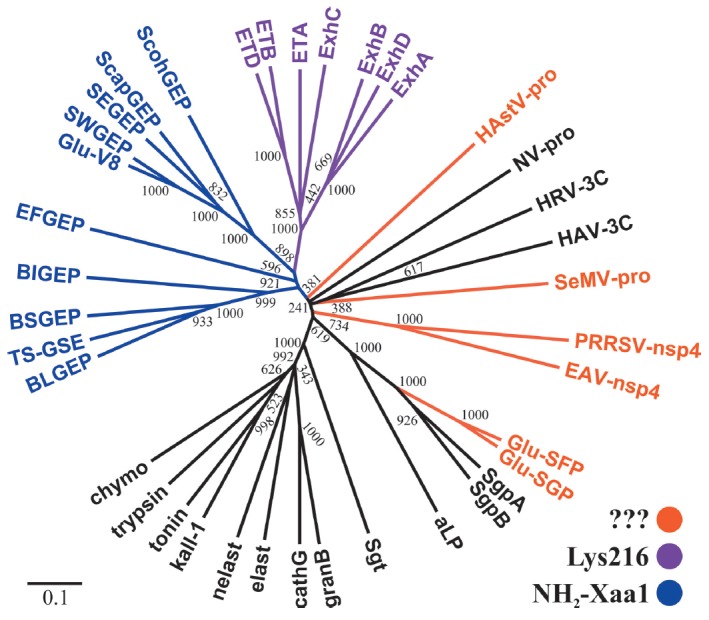
Phylogenetic tree of chymotrypsin-like proteases. Branches corresponding to GEPases are colored: in orange – a compensator of the substrate charge at the enzyme S1 site has not been identified; magenta – Lys216 at the S1 site; and blue – α-amino group of the N-terminal residue at the S1 site. GEPases: Glu-SFP of Str. fradiae, SEGEP of S. epidermidis, SWGEP of S. warneri, ScohGEP of S. cohnii, ScapGEP of S. caprae, BLGEP of B. licheniformis, BSGEP of B. subtilis, TS-GSE of Thermoactimomyces sp., EFGEP of Ent. faecalis; ExhA, ExhB, ExhC and ExhD – epidermolytic toxins A, B, C and D of S. hyicus. NV-pro – Norwalk virus protease; HRV-3C, HAV-3C – proteases 3C of human rhinovirus and hepatitis A virus; aLP - α-lytic protease of Lysobacter enzymogenes; Sgt, SgpA, SgpB – trypsin, proteases A and B of Str. griseus; kall-1, trypsin, nelast, cathG, granB – human kallikrein 1, trypsin 1, neutrophil elastase, cathepsin G and granzyme B; chymo, elast – bovine chymotrypsin A and elastase 1; tonin – rat tonin. Sequence alignment and neighbor-joining tree reconstruction was carried out using ClustalX 2.1 (www.clustal.org). The tree was visualized with the use of FigTree software (tree.bio.ed.ac.uk/software/figtree/). The numbers represent the number of dendrograms in which the individual bifurcations were reproduced during bootstrap sampling of 1,000 trees.

Staphylococcal ETs are the intermediate variant. On the one hand, their precursors are processed heterocatalytically. On the other hand, processing is not related to activity regulation, since it only involves signal peptide deletion. Hence, neither the formation of a functionally active enzyme before processing nor strict activity regulation is required. The variant observed in GEPases from viruses and *Streptomyces *would be suitable here. However, a phylogenetic analysis demonstrates that ETs are most likely to be Glu–V8 paralogues (*Fig. 9*, see discussion below); i.e., these proteins are “engineered” on the same basis as Glu–V8 and employ essentially the same architecture of the S1 site (*Fig. 8*). Meanwhile, unlike Glu–V8, ETs contain no propeptides, being indicative of a different folding mechanism [115], and exhibit a much narrower specificity. They are inactive with respect to most proteins and peptides, which is possibly attained through inserting the Lys216 residue and reducing the volume of the S1 pocket, as well as due to the unusual conformation of the oxyanion hole [83].

In the context of our discussion, it would be interesting to trace the phylogeny of GEPases. The only attempt at a phylogenetic analysis of enzymes belonging to this group was found in a study published 20 years ago [46]. Therefore, in this review we compared the sequences of the characterized GEPases and some CLPs with different specificities in order to build a phylogenetic tree (*Fig. 9*). First, we would like to mention, as the authors of [46] did, that there is an impression that GEPases have appeared in the phylogenetic tree of GLPs at least twice. This is indicated by the presence of two remote branches of bacterial GEPases: one branch contains proteins similar to Glu-V8 and ET, while the second one corresponds to enzymes from *Streptomyces*. (The phylogenetic position of viral proteases is difficult to infer, since the topology of the resulting tree in the portion concerning these proteins is unreliable.) It is especially illustrative that GEPases from *Streptomyces *are just a small sprout in the branch of bacterial proteases exhibiting broad specificity. This observation gives grounds for assuming that there is quite a high probability that glutamate specificity (actually, any other specificity) develops via the chymotrypsin fold. Modification of the key residues of the S1 pocket (His213, Thr/Ser190) that provide the required geometry and minimal interactions for the binding of Glu/ Gln residues is apparently needed for that. However, this basic specificity probably needs to be enhanced, which can be achieved through different mechanisms, in particular by inserting a compensator into the S1 site. However, this is not the only possibility as demonstrated by the analysis of enzymes from viruses and *Streptomyces*. Special attention should be focused on the branch combining all bacterial GEPases, except for enzymes isolated from *Streptomyces*. As expected, the topology of this branch corresponds to the taxonomy of producer bacteria. ETs and staphylococcal enzymes, such as Glu-V8, share the phylogenetic tree’s branch; i.e., they are structurally closer to each other than they are to the remaining bacterial GEPases.

## CONCLUSIONS

Our analysis demonstrates that all known GEPases belong to the structural family of chymotrypsin and possess a similar overall structure of the S1 substrate-binding site. Enzymes in this group have several different systems of substrate charge compensation. The differences in the mechanisms of negative charge recognition correlate with the differences in the architecture and processing pathways of the precursors, which is probably determined by the biological functions of the corresponding proteases. All these facts provide grounds for assuming that GEPases have emerged in the phylogenetic tree of CLP at least twice. However, we have to admit that the data on the structure and mechanisms of action of GEPases available today are not sufficient to solve the puzzle of their strict substrate specificity.

It should be emphasized that the focus of studies devoted to GEPases shifts from the investigation of enzymes towards analyzing their biological functions, typically because of the pathogenesis. Thus, the involvement of staphylococcal GEPases in the regulation of biofilm growth is studied intensively today, primarily due to the hope of finding new strategies to combat staphylococcal infection [116]. Viral GEPases are being thoroughly studied in connection with attempts to design effective antiviral drugs. Meanwhile, GEPases are usually not isolated from the entire pool of 3C-like proteases in pursuit of universal inhibitors of the processing of viral polyproteins. Engineering of inhibitors requires extensive investigation into protein–ligand interactions, which implies obtaining a large body of structural data (e.g., [76, 117]). The study of the role of GEPases in the viral life cycle is still underway [24, 118]. The recent studies devoted to viral 3C and 3C-like proteases, including GEPases, as apoptosis inductors deserve special mention [119-122]. Research into GEPases in the medical context will undoubtedly continue. It should be emphasized that, since strict substrate specificity underlies the biological activity of GEPases, novel data on its structural determinants will be inevitably collected during these studies.

## Abbreviations

3Cpro: picornaviral 3C protease
3CLpro: 3C‑like protease
3CLSP: 3C-like serine protease;
BIGEP: GEPase of B. intermedius
Boc-AAPE: tert-butyloxycarbonyl-Ala-Ala-Pro-Glu
CLP: chymotrypsin-like protease
EAV-nsp4: nonstructural protein 4 of equine arteritis virus
Esp: extracellular serine protease of S. epidermidis
ET: epidermolytic toxin
ETA, ETB: epidermolytic toxins A and B of S. aureus
GEPase: glutamyl endopeptidase
(+)RNA-virus: positive-sense single-stranded RNA virus
Glu‑SGP: GEPase of Str. griseus
Glu/Gln‑P1: an amino acid residue at position P1 of a substrate
Glu‑V8: protease V8 of S. aureus
HAstV‑pro: human astrovirus protease
PDB ID: Protein Data Bank (http://www.rcsb.org) identifier
PRRSV‑nsp4: nonstructural protein 4 of porcine reproductive and respiratory syndrome
SeMV‑pro: Sesbania mosaic virus protease
